# Beyond Acute Overdoses: A Case of Chronic Clozapine Toxicity With Non-specific Presentation

**DOI:** 10.7759/cureus.79771

**Published:** 2025-02-27

**Authors:** Shahzad Dildar, Muhammad Ali Javaid

**Affiliations:** 1 Care of the Elderly, Prince Charles Hospital, Cwm Taf Morgannwg University Health Board, Merthyr Tydfil, GBR

**Keywords:** atypical antipsychotics, atypical antipsychotics toxicity, chronic atypical antipsychotics toxicity, chronic clozapine toxicity, clozapine, clozapine side effects, clozapine toxicity, second generation antipsychotics, second-generation antipsychotics toxicity

## Abstract

Clozapine is recognized as the gold standard for treating treatment-resistant schizophrenia but carries a notable risk of severe adverse effects. This case report presents a 61-year-old Welsh woman with paranoid schizophrenia and epilepsy who, while on long-term clozapine and valproic acid therapy, developed a progressive decline in mobility, cognition, and social engagement, punctuated by recurrent falls. Despite numerous hospital visits and extensive investigations, the underlying cause of her symptoms remained elusive until serum clozapine level testing revealed toxicity, with levels significantly exceeding the therapeutic range. Following the gradual tapering and ultimate cessation of clozapine, the patient exhibited marked improvements in both mobility and cognitive function. This case highlights the potential utility of serum level monitoring in patients on clozapine, especially those showing unexplained deterioration in mobility and social engagement, as toxicity can arise even after prolonged stable treatment. The findings emphasize the necessity for vigilant monitoring and proactive management strategies to mitigate the risk of severe neurological complications associated with clozapine therapy.

## Introduction

Clozapine is considered the gold standard treatment for treatment-resistant schizophrenia [1]. Alterations in consciousness, weight gain, constipation, sialorrhea, orthostatic hypotension, and tachycardia had been observed in 10% to 50% of patients, while fever, seizures, agranulocytosis, myocarditis, cardiomyopathy, neuroleptic malignant syndrome, and urinary incontinence had occurred rarely, affecting 1% to 10% of patients [2]. A fatal outcome can occur in 25% of patients who develop rare adverse effects [3]. Our case reports the incidence of recurrent falls, declined mobility, and social withdrawal along with loss of appetite and self-care while on long-term clozapine monotherapy for treatment-resistant schizoaffective disorder without signs and symptoms of acute intoxication.

Written informed consent for publication of the clinical details and/or images was obtained from the patient's sister, who had the lasting power of attorney, as the patient lacks mental capacity. 

## Case presentation

A 61-year-old Welsh female diagnosed with paranoid schizophrenia presented to Accident and Emergency following a non-mechanical fall and slow-onset confusion. She described falling from a standing height and experiencing dizziness but reported no preceding symptoms such as light-headedness, palpitations, or weakness. The patient expressed concern to the attending physician, stating, “I want to know why I am falling.” However, she struggled to clearly recall her usual activity level and mobility status prior to the onset of recurrent falls. She resides in a sheltered accommodation with daily visits from a carer and is managed for epileptic seizures with oral valproic acid.

**Figure 1:**
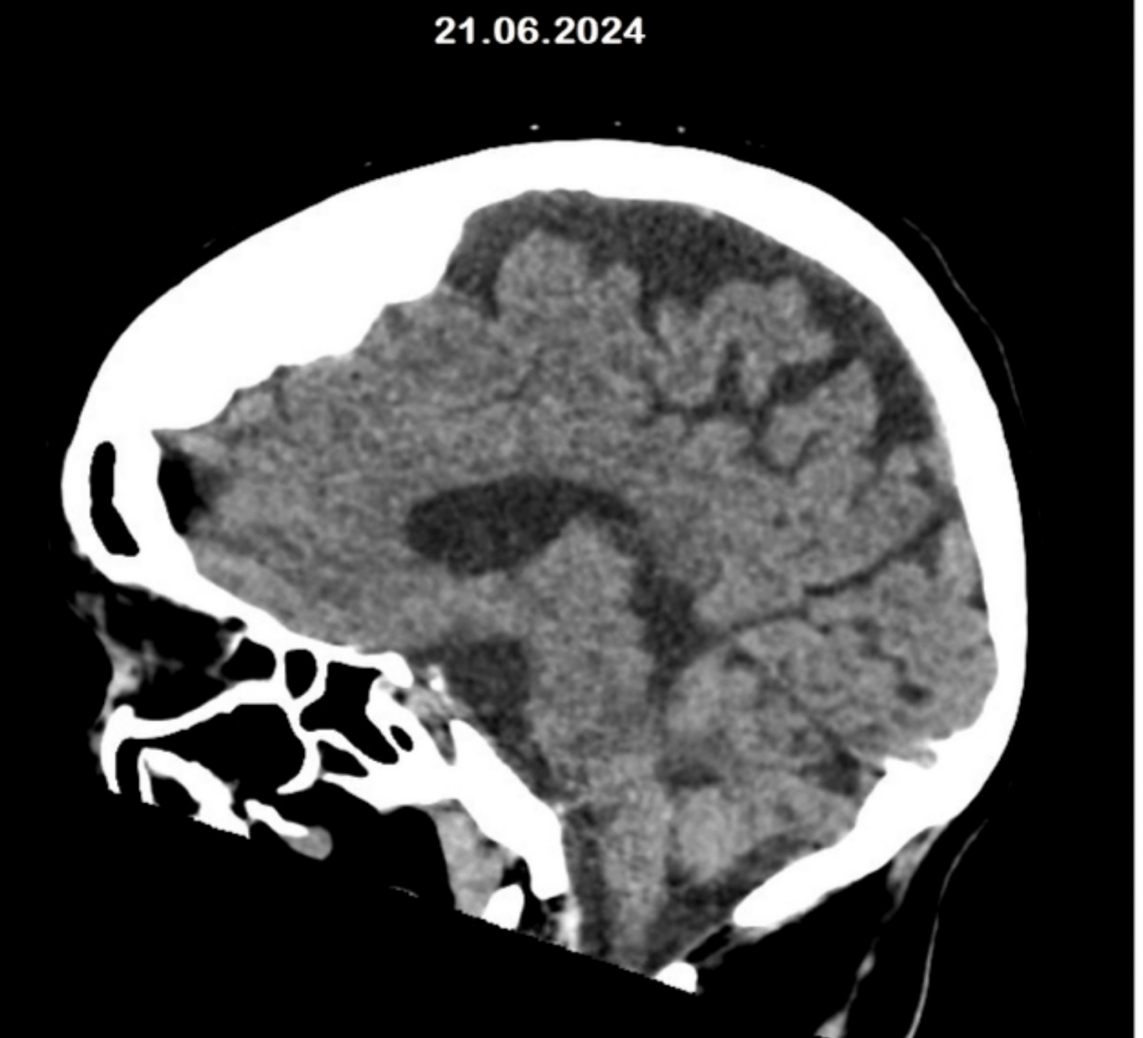
Non-contrast CT scan of the head. A non-contrast CT scan of the head was done at the time of admission as part of the frailty and confusion screen.

Over the past six months, she has experienced multiple unprovoked recurrent falls, leading to eight visits to Accident and Emergency. Each visit involved investigations, including blood tests (Table 1) and computed tomography (CT) scans (Figure 1), as part of a frailty and confusion screening. Despite the absence of an explanation for her falls, she was discharged back to her sheltered accommodation on the same day after each visit.

**Table 1:** Frailty and confusion screening blood test panel. *ug/L: micrograms per liter; g/L: grams per liter; L/L: liters of red blood cells per liter of blood; fL: femtolitre; pg: picogram; mg/L: milligrams per liter; mmol/L: millimoles per litre; U/L: units per liter; nmol/L: nanomoles per litre; mU/L: milliunits per litre; pmol/L: picomoles per liter; ng/L: nanograms per liter; ug/L: micrograms per liter. Clozapine value marked in bold.

Test	Result	Units	Normal Range
Hemoglobin (Hb)	127	g/L	(115-165)
White blood cell (WBC) count	6.9	x10^9/L	(4.0-11.0)
Platelet (PLT) count	249	x10^9/L	(150-400)
Red blood cell (RBC) count	4.58	x10^12/L	(3.80-5.50)
Hematocrit (Hct)	0.40	L/L	(0.37-0.47)
Mean cell volume (MCV)	88	fL	(80-100)
Mean cell hemoglobin (MCH)	27.7	pg	(27.0-33.0)
Red cell distribution width (RDW)	14.4	%	(11.0-14.8)
Neutrophil count	4.6	x10^9/L	(1.7-7.5)
Lymphocyte count	1.3	x10^9/L	(1.0-4.5)
Monocyte count	1.0	x10^9/L	(0.2-0.8)
Eosinophil count	0.0	x10^9/L	(0.0-0.4)
Basophil count	0.0	x10^9/L	(0.0-0.1)
Nucleated red blood cell (NRBC) count	0.0	x10^9/L	
C-reactive protein (CRP)	<1	mg/L	(<5)
Magnesium	0.84	mmol/L	(0.70-1.00)
Sodium	143	mmol/L	(133-146)
Potassium	4.1	mmol/L	(3.5-5.3)
Urea	5.9	mmol/L	(2.5-7.8)
Creatinine	46	umol/L	(46-92)
Estimated glomerular filtration rate (eGFR)	>90	ml/min/1.73m²	
Creatine kinase	120	U/L	(25-200)
Lactate dehydrogenase	182	U/L	(<250)
25-hydroxyvitamin D (immunoassay)	146	nmol/L	
Thyroid-stimulating hormone (TSH)	3.39	mU/L	(0.27-4.20)
Free tetraiodothyronine (T4)	14.7	pmol/L	(11.0-25.0)
Vitamin B12	836	ng/L	(180-900)
Folate	>20.0	ug/L	(>3.0)
Ferritin	85	ug/L	(15-300)
Valproic acid	22	mg/L	

In addition to the increasing frequency of falls, she developed new-onset overflow urinary incontinence and action tremors. Her sister reported a sharp decline in her general activities and social engagement during this period. Previously independent, she had never used a walking aid and frequently visited local tourist destinations by taxi.

Further inquiry with her sheltered residential home manager revealed that she began dragging her leg and exhibited a generalized decline in both mobility and appetite over the past six months. Her general practitioner (GP) noted the development of a short gait and a tendency to lean to her left side while walking. Prior to this admission, she was able to use the washroom independently; however, as an inpatient, she became bedbound and reliant on a bedpan. Her sister reported worsened sleepiness since hospitalization. She sustained another non-traumatic fall in the hospital while attempting to transfer independently from bed to chair, describing it as sliding off the bed to the floor. During her hospital stay, she stopped eating independently, although she could tolerate food if assisted. A nasogastric tube was placed for nutritional support upon the dietitian's advice.

Despite thorough investigations, no abnormalities were found to explain her symptoms. The mental health liaison team recommended serum clozapine level testing. Due to the infrequent practice of clozapine serum level testing due to inconsistencies in serum clozapine levels and its therapeutic effects, samples had to be sent to the regional laboratory. Results showed serum clozapine levels at 1627 ug/L, exceeding three times the therapeutic window of 350-600 ug/L. Consequently, two doses were omitted over the next 24 hours, and her clozapine dosage was titrated down over two weeks. Repeat serum levels subsequently returned to be within the therapeutic range. Based on the psychiatrist's advice, clozapine was gradually discontinued, as she exhibited no signs of paranoid schizophrenia at subtherapeutic doses. Eventually, she was entirely tapered off clozapine.

**Figure 2:**
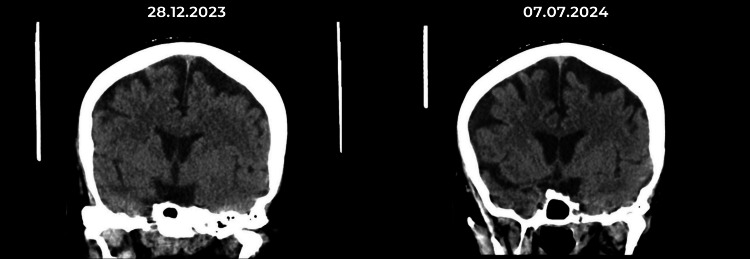
Non-contrast CT scans of the head. Both CT scan heads (non-contrast) were done six months apart. The image illustrated on the left was done on December 28, 2023, while the one on the right was done on July 7, 2024.

A review of her CT head scan (Figure 2) done six months apart revealed worsened cerebral atrophy, prompting the neurology team to switch her medication from valproic acid to lacosamide to protect against further cerebral pseudoatrophy associated with valproic acid. The neurophysiologist concluded that the electroencephalogram (EEG) showed a few sharp waves and slow wave changes noted over the temporal regions, but not enough to account for the change in her behavior. The EEG is degraded by muscle activity throughout. However, no overt epileptiform activity was present.

Following the cessation of clozapine, her mobility and cognition significantly improved within a short period. She removed the nasogastric tube and regained her appetite, verbally requesting her favorite meals. During an assessment, she remarked to a doctor, “I feel like I was sleeping for a very long time, but now I am awake.” She began interacting with the nursing staff and other patients on the ward. Her mobility improved to the point where she could use a wheeled walking frame, initially with assistance and eventually independently. Although her gait improved from a festinating one to a normal one, she remained unsteady and was advised by the physiotherapist to continue using the wheeled walking frame. Ultimately, the patient was discharged back to her sheltered residential home.

## Discussion

Schizophrenia is a neuropsychiatric disorder marked by disturbances in perception, cognition, and behavior, characterized by delusions and hallucinations. Neurotransmitter abnormalities involving dopamine, serotonin, glutamate, and gamma-aminobutyric acid (GABA) are hypothesized to play significant roles in the pathophysiology of schizophrenia. The link between dopamine and schizophrenia was discovered through the serendipitous finding that dopamine D2 receptor antagonists effectively alleviate psychotic symptoms. Treatment-resistant schizophrenia is defined as an inadequate response to at least two neuroleptic antipsychotics at optimal doses; 20%-45% of individuals having schizophrenia for over two years were only partially responsive to antipsychotic medication [4]. Schizophrenia has a lifetime prevalence of 0.48% [5]. Clozapine is the drug of choice in treatment-resistant schizophrenia.

Clozapine is an atypical antipsychotic classified as a dibenzodiazepine antipsychotic that acts as a receptor antagonist for dopamine receptors (preferentially D4 receptors over D2 receptors), serotonin receptors, muscarinic receptors (M1, M2, M3, M5), histamine receptors, and alpha-1 adrenergic receptors, while also acting as a partial agonist at 5-hydroxytryptamine (5-HT)1A receptors. Clozapine’s metabolite norclozapine acts as an allosteric modulator on M1 and M4 receptors [6]. Despite this peculiar receptor modulation profile and the advantage of decreasing suicide risk associated with the use of antipsychotics, the therapeutic applications of clozapine are restricted because of the associated potentially severe or even life-threatening adverse effects [3].

The pharmacokinetics of clozapine are intricate, as they involve multiple cytochrome P450 enzymes. This complexity can lead to significant variability in serum levels and an elevated risk of adverse effects, especially when clozapine interacts with other substances. Certain medications and commonly consumed items, such as caffeine, can increase clozapine serum levels by inhibiting the cytochrome P450 1A2 (CYP1A2) pathway [7]. This interaction necessitates careful monitoring and potential dose adjustments to ensure both efficacy and safety in treatment. During acute infections or chronic inflammatory states, pro-inflammatory cytokines, especially interleukin-6 (IL-6), can significantly downregulate cytochrome P450 enzyme activity by up to 90%, leading to elevated serum levels of clozapine and an increased risk of toxicity [8]. Due to its complex metabolism, there are considerable inter- and intra-individual variations in clozapine serum levels for a given dose; however, the range of serum levels associated with toxicity remains unclear, and while central nervous system side effects may correlate with serum levels, many adverse effects of clozapine, such as hematological and cardiac events, seem to be unrelated [9].

According to Montastruc et al., clozapine was the third most lethal drug worldwide from 2010 to 2019, with 1,860 fatal outcomes, of which 968 cases occurred in the United Kingdom [10]. The major causes of fatal outcomes in patients treated with clozapine included pneumonia (relative lethality= 30%), sudden death (relative lethality=90%), agranulocytosis (relative lethality=2%), myocarditis (relative lethality=12%), constipation (relative lethality=12%), arrhythmia (relative lethality=5%), seizures (relative lethality=5%), and syncope (relative lethality=7%) [11]. In a prospective-observational study, 21.8% of patients started on clozapine developed bradykinesia, while tremors were seen in 24.4% of patients, and only 5.6% exhibited symptoms of akathisia [12]. In another combined retro- and prospective study of extrapyramidal side effects, 33% of the patients started on clozapine showed symptoms of parkinsonism (mainly bradykinesia), 3% developed tremors, and 7% had akathisia [13].

Antipsychotics are the most common cause of drug-induced parkinsonism (DIP), typically manifesting within a few days or weeks after starting treatment, though in rare cases, onset can be delayed by several months or more [14]. The neurological adverse effects of clozapine can be attributed to dysfunction of adenosine receptors, blockade of dopaminergic type D2 receptors, supersaturation of dopaminergic type D2 receptors, dysfunction of the basal ganglia of the thalamocortical motor loop, melatonin metabolism disorder, violation of vitamin D3 levels, and oxidative stress [15]. Primarily, antagonism of dopamine D2 receptors in the striatum disinhibits GABA neurons, enkephalin, and the subthalamic nucleus in the indirect pathway, leading to increased GABA inhibition in the thalamocortical projection, causing a relative decrease in the activity of thalamocortical circuits, resembling the motor loop impairment similar to that seen in Parkinson's disease [14]. Radioactive clozapine demonstrates rapid and transient occupancy of dopamine D2 receptors, dissociating in under 60 seconds (in comparison, haloperidol and chlorpromazine have receptor occupancy of 30 minutes), which contributes to the clinical effectiveness of atypical antipsychotics [16]; however, at chronic toxic doses, this rapid dissociation may diminish, leading to movement disorders similar to Parkinson's disease. Drug-induced parkinsonism symptoms usually resolve within weeks to months after stopping the offending drug; however, parkinsonism may persist or progress in 10-50% of patients [14].

## Conclusions

Clozapine is the mainstay of treatment for treatment-resistant schizophrenia. Clozapine is found to be more efficacious; however, due to associated unexpected adverse effects and high fatality, its use is limited. Clozapine toxicity may not always be so obvious or discrete. Clozapine monitoring in the United Kingdom is emphasized in hematological clinics to watch out for agranulocytosis only. Due to the rarity of occurrence of long-term clozapine toxicity, unexplained decline in mobility, and drop in social engagement, it should prompt investigation into clozapine toxicity alongside other investigations. While serum clozapine levels don't reliably predict therapeutic efficacy, they remain a valuable tool for monitoring potential toxicity. The close correlation between serum levels and neurological adverse effects makes monitoring crucial for early detection and intervention. Patients even stable with clozapine use for years may develop toxicity later. Inconsistent clozapine adherence significantly increases the risk of toxicity. Clinicians must maintain heightened vigilance and a low threshold for suspicion in patients struggling with regular medication use. Furthermore, cytochrome P450-dependent metabolism can cause serum level fluctuation even in patients on long-term clozapine; a high index of suspicion in patients on clozapine could help early detection, e.g., smoking, selective serotonin reuptake inhibitors (SSRIs), and proton pump inhibitors (PPIs) can increase or decrease serum clozapine levels by virtue of down-regulating or up-regulating the cytochrome P450 system.
